# Health Economic Evaluation of Varicella Vaccination Within the Swedish National Vaccination Programme for Children

**DOI:** 10.1111/apa.70506

**Published:** 2026-03-26

**Authors:** Frida Kasteng, Gianpaolo Scalia Tomba, Annika Ersson, Ellen Wolff, Sören Andersson, Kari Johansen

**Affiliations:** ^1^ Department of Public Health Analysis and Data Management Public Health Agency of Sweden Solna Sweden; ^2^ Department of Mathematics University of Rome Tor Vergata Rome Italy; ^3^ Department of Mathematics Stockholm University Stockholm Sweden

**Keywords:** catch‐up vaccination, cost‐utility analysis, epidemiological modelling, immunisation strategies, varicella vaccine

## Abstract

**Aim:**

To assess the cost‐effectiveness of including varicella vaccination in the Swedish national vaccination programme for children together with the added impact of catch‐up vaccination of susceptible older children.

**Methods:**

An epidemiological transmission model was used to assess the cost‐effectiveness of a two‐dose national varicella vaccination programme, with and without a catch‐up strategy, compared to no varicella vaccination. Parameter estimates were based on scientific publications and data from Swedish national and regional registries.

**Results:**

A national varicella vaccination programme would result in health gains and cost‐savings from a societal perspective. Cost‐savings were primarily due to a reduction in caregiver productivity loss. From a health system perspective, the cost per quality‐adjusted life‐year gained was estimated at around SEK 200 000 (EUR 18 000), both with and without catch‐up vaccination. At a negotiated vaccine price reduction to 30% of the current list price, the cost per quality‐adjusted life‐year gained would come down to SEK 5 000 (EUR 450).

**Conclusion:**

Our analyses suggest that inclusion of varicella vaccination in the national vaccination programme for children would be a cost‐saving strategy from a societal perspective, which together with a catch‐up vaccination offer for susceptible older children would result in a fast decline of varicella in Sweden.

## Summary

- An economic evaluation was carried out to assess the cost‐effectiveness of including varicella vaccination in the national vaccination programme for children in Sweden.
- Child vaccination against varicella in Sweden would be a cost‐saving intervention from a societal perspective, resulting in both health gains and reduced costs.
- A high‐coverage programme would stop varicella disease transmission within a few years' time, even faster if combined with catch‐up vaccination for susceptible older children.

**Abbreviations**
HZ: herpes zoster
ICER: Incremental cost‐effectiveness ratio
MMR: measles, mumps and rubella‐containing combination vaccine
MMRV: measles, mumps, rubella and varicella‐containing combination vaccine
NIP: National immunisation programme
QALY: Quality‐adjusted life‐year
V: monovalent varicella‐containing vaccine
VZV: varicella zoster virus

## 1 Introduction

Varicella (chickenpox) is the primary infection caused by the highly contagious varicella zoster virus (VZV). The virus commonly causes skin lesions that typically last for 5–7 days. In Sweden, incidence peaks at around 4 years of age and a majority of children are infected before reaching adolescence [1]. Most children do not need to seek medical care as the infection is often mild. However, around 0.2% of children with varicella in Sweden require hospitalisation due to complications [2]. Furthermore, a fourfold increased risk of stroke has been measured in the time period up to 6 months after an acute primary VZV infection in children [3]. An average of eight individuals per year in Sweden died with varicella as a contributing factor during the period 2013–22. Of these, on average, one death per year affected a child under the age of 15 while 5 occurred in individuals aged 65 or older [2]. Chickenpox in adolescents and adults usually leads to a higher rate of debilitating symptoms and complications [2].

After infection with VZV, the virus remains latent in the nervous system. If the immune system is weakened later in life due to age or suppressed because of disease or medication, the virus can be reactivated and cause herpes zoster (HZ) (shingles). Subsequent exposure to the VZV in individuals who have already had varicella might reduce susceptibility to HZ, so‐called exogenous boosting [4].

The first varicella vaccine was developed in Japan in the 1970s [5]. The USA was the first country to introduce a universal vaccination programme for varicella in 1995 [6]. Population studies in the USA have not reported any serious adverse effects of the used monovalent and tetravalent vaccines [7]. A first dose provides an estimated 81% (95% CI 78–84) protection with some waning over time while a second dose provides a 92% (95% CI 88–94) protection that remains stable over time, based on available follow‐up data on post‐licensure estimates of vaccine effectiveness [8]. In the Nordic countries, national child immunisation programmes (NIPs) against chickenpox have been in place since 2017 in Finland and since 2020 in Iceland. Several other European countries have included varicella vaccination in their NIPs, including Germany, Italy and Spain [9].

The purpose of this study was to assess the cost‐effectiveness of including varicella vaccination in the NIP for children in Sweden, together with a catch‐up for older non‐immune children, motivated by epidemiological and ethical reasons to reduce disease transmission as fast as possible upon the initiation of a vaccination programme. The results of the analysis have previously been submitted to the government of Sweden [10], and have informed the decision to include varicella vaccination in the NIP for children in Sweden from 2027.

## 2 Methods

### 2.1 Setting and Perspective of the Analysis

We carried out a health economic analysis to assess the cost‐effectiveness of including vaccination against varicella in the NIP for children in Sweden. The results are presented as cost per quality‐adjusted life year (QALY) gained, also commonly referred to as the incremental cost‐effectiveness ratio (ICER) in health economic analyses. The results are presented from both a societal and a health care system perspective [11].

The analysis compares two intervention scenarios with a control scenario. In the first scenario, varicella vaccination is provided to young children, the first dose at age 18 months and the second dose at age 7 years. As a second scenario, a catch‐up strategy targeting older children with no known history of varicella infection based on caregiver recall is added to the first scenario. The control scenario is a setting without any varicella vaccination in the population.

The parameter estimates in the model were based on scientific publications, data from Swedish national and regional registries, and national treatment guidelines [2, 12, 13, 14]. In cases where published data were missing, assumptions from a Swedish expert group invited by the Public Health Agency of Sweden were used [10].

### 2.2 Epidemiological Model

As basis for the health economic analyses, we used a deterministic, compartmental, age‐structured, dynamic transmission model developed in the C programming language [14, 15]. The model was an extended age‐dependent SIR (susceptible, infected, recovered) model, in which individuals moved in age and between different health states depending on age‐specific risk of disease. The flow between health states is illustrated in Figure 1. In each model cycle, individuals could move from one health state to another, stay in the same health state, or die. Movements between health states were defined as differential equations. The cycle length was 1 day, and the modelled population was assumed to be stable over the modelled time horizon. A constant birth rate of approximately 120 000 children per year was assumed. Model outflow was based on natural mortality [13]. The epidemiological modelling of different varicella immunisation strategies in Sweden are presented in a separate article [16]. Further details on the mathematical formulation of the model and the fitting to varicella and HZ incidence data can be found in the supplementary data of an earlier publication [14].

**FIGURE 1:**
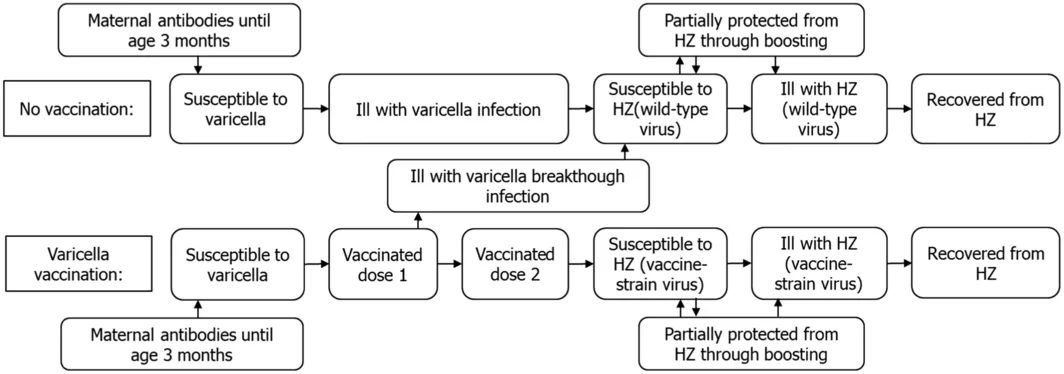
Flowchart of the epidemiological model.

Protection from maternal antibodies was assumed until 3 months of age, after which susceptibility to VZV was expected. Force of infection and contact rate were calibrated to fit Swedish seroprevalence data for children up to age 12, matched with Finnish data for older age groups [1, 17]. Contact patterns were based on a synthetic matrix that described the intensity of total contacts between age groups [18]. VZV incubation time was set at 14 days in the model. The illness duration and infectious period (at a constant rate) was 7 days. After recovery from infection with VZV, individuals became susceptible to HZ. The reactivation rate of HZ was age‐dependent and assumed to occur only once. Individuals with HZ transmitted the disease only to a very limited degree in the model. The model allowed for so‐called exogenous boosting, that is, an individual susceptible to HZ who was exposed to the VZV would gain a degree of protection against the incidence of HZ [14].

Vaccinated children received protection against infection corresponding to the effectiveness of the vaccines [8, 19, 20]. To include all relevant effects of the vaccination programme, the time horizon was set at 95 years. This allowed the model to account for long‐term protection of varicella vaccination against HZ. The vaccine strain of VZV is less likely to result in HZ later in life compared with wild‐type VZV, as well as leading to a less severe form of HZ [21, 22].

### 2.3 Economic Evaluation

For the health economic calculation, data from the epidemiological model were extracted to Excel. The output from the epidemiological model groups the number of individuals in each health state on a yearly basis (0–95) by age group (one‐year groups for ages 0–14 years; 10‐year groups for ages 15–94). To perform the health economic analyses, the data were matched with the corresponding resource use, the costs of resource use, and the quality‐of‐life impact.

Results were calculated for both a societal and a health system perspective. Reporting standards for health economic analyses were used as guidance for the presentation of the results [11]. One‐way and two‐way sensitivity analyses were carried out to assess the sensitivity of results to variations in key input variables.

The healthcare cost data used in the model are presented in 2023 SEK values. Both health effects and costs were discounted by 3% annually, but are also presented without discounting in the sensitivity analysis [23].

There is no explicit threshold for when an intervention is considered to be cost‐effective in Sweden [24]. The cost per QALY framework against which we present our results in this report was based on the methods guidelines from the National Board of Health and Welfare where less than SEK 100 000 is considered a low cost per QALY and SEK 100 000–499 999 a moderate cost per QALY [25].

### 2.4 Parameters and Assumptions

#### 2.4.1 Incidence of Varicella and HZ

Varicella incidence was based on a study of 957 blood samples from children aged 0–5 years and 12–13 years during the period 2011–2013 [1]. Data from Finland, collected in 1997–1998, were used to estimate seroprevalence in older age groups [17]. It is possible that migration has affected the seroprevalence in later years; however, the number of hospitalisations has remained similar over time and could serve as a proxy for these seroprevalence data still being valid.

We include HZ incidence in the model due to the impact of varicella vaccination on future HZ susceptibility in vaccinated individuals and the effect of exogenous boosting from VZV on HZ susceptibility.

#### 2.4.2 Impact of Intervention

The effectiveness of varicella vaccination on all varicella was set at 81% for the first dose with a 2% annual waning rate, and at 92% for the second dose with no waning effect following the second dose [8, 19, 26]. The vaccine effectiveness against moderate/severe varicella has been estimated at 98% after the first dose [8]. In or analyses, this was accounted for in terms of the probability of hospitalisation and specialist care need for breakthrough episodes. We assumed the same effectiveness against varicella for both monovalent as well as the tetravalent vaccines [26, 27]. Varicella infection following vaccination was referred to as breakthrough infection and assumed to be mild, for example, not incurring a risk of specialised or inpatient care in the model. We modelled risk of HZ in vaccinated individuals over the 95‐year time horizon of the model, the HZ reactivation risk with the vaccine strain virus was set at 10% of the risk with wild‐type viruses [21, 22].

Vaccination coverage was assumed to be 95% for both the national vaccination programme and the catch‐up vaccination, for both doses [28]. This assumption was varied in sensitivity analyses.

The model accounted for herd immunity effects of vaccination, since the force of infection is proportional to both the number of infected and susceptible individuals. Herd immunity occurs when there are too few susceptible (because of high coverage of vaccination) to sustain disease transmission.

Exogenous boosting refers to protection against HZ due to immune system stimulation after re‐exposure to VZV in already infected individuals. In the base case analysis, exogenous boosting is assumed to fully prevent HZ during 4 years following exposure to VZV among seropositive individuals [14]. Exposure to VZV depends on the prevalence of infection in the population. The impact on the cost‐effectiveness results of the alternative assumptions about impact of boosting is explored in the sensitivity analyses [4, 14].

### 2.5 Resource Use and Costs

We modelled a national varicella vaccination programme where the first dose is administered at 18 months of age, during an already existing visit to the child health services, and the second dose at age 7 years, by the school health services, at the same time as the measles, mumps and rubella (MMR) vaccine. As both visits are already scheduled for vaccine administration in the national vaccination programme for children with another vaccination given at the same time, we assumed that administration costs would encompass only the extra nurse time required for vaccine administration (including the preparation of the vaccine and syringe, thorough vaccine information about effects and side effects, addressing parents' questions, pain reduction, and the subsequent documentation in the child's record), not the costs of a full visit. This was assessed as an additional 15 min of a school nurse's time per administered dose, based on the average gross salary for school nurses in 2022 [13, 29]. Catch‐up vaccinations were assumed to occur primarily during already scheduled visits at the child health services and/or the school health services, up to the age of 12 years in the model. The administration cost did not include the cost for facilities or overheads.

The vaccination cost applied in the base case analysis was the average list price per dose of the two monovalent vaccines currently available in Sweden (Varilrix at SEK 483.50 and Varivax at SEK 488.50) [30]. As sensitivity analyses, we assessed the second monovalent dose being provided at 5 years of age as well as the use of one of the tetravalent MMRV vaccines for the second dose if administered at age 7 years. Since neither of the two MMRV vaccines are listed in Sweden, we used the average price for the European region from the WHO Mi4A Vaccine Purchase Database [31], approximately SEK 780 (USD 1 = SEK 10.6—average exchange rate 2023 [32]), minus the current list price of the MMR vaccine in Sweden (SEK 124) [30], with no additional administration cost assumed in the analysis since no extra shot was being administered during the visit.

Healthcare need as a consequence of primary VZV infection was based on a study on the burden of varicella in Sweden published in 2016 where resource use data constituted the average consumption during the period 2007–2012 [33]. Although these data are a few years old, we chose to use them as they matched the time period of the most recent seroprevalence data from Sweden [1]. To validate its use in the model, we compared average annual varicella cases for the period 2007–2012 with the time period 2014–2019 [2]. Admission rates were relatively similar, while specialist consultations were 40% higher during the second time period. The differences may be due to different epidemic profiles during the two time periods, and thus it is arguably more accurate to use resource use data that are closest in time with the available seroprevalence data. Admissions and specialist care visits were approximately halved due to COVID−19 restrictions in the years 2020–2021 and remained at a lower level than before the pandemic in 2022; thus, data from the three most recent years were not deemed representative for the long‐term perspective of the analysis [2]. We included all hospitalisations with varicella as either primary or secondary diagnosis in the analysis, as advised by the external reference group [10].

HZ incidence, primary healthcare need, pharmaceutical prescription and risk of persisting pain following HZ were based on Swedish studies published in 2013 and 2015 using data from 2008 to 2010 [34, 35]. The number of primary care visits for HZ and persisting pain following HZ was based on secondary data [15, 36].

In the model, we used in‐patient and specialist out‐patient care use for HZ from the National Patient Register for the corresponding time period [2]. We only included hospitalisation with HZ as the primary diagnosis in the calculations, as advised by the external reference group [10].

For both varicella and HZ, the unit costs of primary care visits, specialised out‐patient care, and hospitalisations were based on the ‘Cost‐per‐patient’ database [37]. Average costs over the years 2019–2021 were updated to 2023 values.

We calculated the total cost for varicella and HZ by multiplying the average cost per visit/admission by the average number of visits/admissions per patient in each group. These data points were extracted from the online database of diagnoses in in‐patient and specialised open care provided by the National Board of Health and Welfare [2]. Information on pharmaceuticals prescribed was derived from national guidelines [12].

Indirect costs were included in the analysis as productivity losses in case of illness. The occupational rates by age group were based on year 2022 statistics [13]. The cost of productivity losses was calculated on the basis of the average gross monthly salary in 2022 [13]. For varicella, information on caregiver productivity loss was derived from days of caregiver leave reported to the Swedish Social Insurance Agency. We used the average annual rates over the period 2011–2019 [38]. A division of this data with the annual number of cases per year based on the incidence rates from the epidemiological model indicates that caregiver leave was reported for on average 41% of estimated incident cases in children over the period 2011–2019 (range 31%–51%) and that those caregivers stayed at home 3.4 days on average. For older individuals with varicella and sick leave due to HZ, the days of illness were based on secondary data [39].

The QALY reduction due to varicella was based on estimates from British studies [40, 41]. We validated the average QALY loss assumptions for all varicella applied on our model against estimates from a study that assessed the QALY loss per case in need of paediatric emergency care or hospitalisation at 0.0055 (2.0 net days) and 0.0265 (9.8 net days) respectively [42]. Since medical care is often not required for varicella, less than 10% of cases in Sweden seek primary care and hospitalisations range to a few per thousand incident cases, the QALY loss we applied across all varicella of 0.0027 was deemed reasonable under the assumption that children not requiring medical care had a QALY loss about half as large as those who sought paediatric emergency care.

The average duration and proportional utility loss due to different degrees of pain associated with HZ and the burden of persisting pain following HZ was also derived from British studies [43, 44]. This percentile utility loss was multiplied with the age‐adjusted utility for the general population in Sweden [10, 45].

### 2.6 Assessment of Uncertainty

In order to investigate the robustness of the results from our analysis, we conducted several deterministic sensitivity analyses. The parameters varied in the sensitivity analyses are presented in Table 1.

**TABLE 1:** Model parameters.

Parameter	Base case values	Alternative values in sensitivity analyses
*Vaccination programme*		
Target population national programme	120 000 children	Not altered
Timing of vaccination	Dose 1: Age 18 months Dose 2: Age 7 years	Dose 1: Not altered Dose 2: Age 5 years
Vaccination programme coverage [28]	Dose 1: 95% Dose 2: 95%	Dose 1: 75%, 85% Dose 2: 75%, 85%
Type of vaccine, cost [30, 31]	Dose 1: Monovalent VAR, SEK 486 Dose 2: Monovalent VAR, SEK 486	Dose 1: Monovalent VAR, SEK 486 Dose 2: Tetravalent MMRV; SEK 656^a^ if dose 2 at age 7
Vaccine effectiveness all varicella [8] Vaccine effectiveness moderate/severe disease [8]	Dose 1: 81% Dose 2: 92% Dose 1: 98%	
Vaccine effectiveness HZ [21, 22]	10% the HZ risk of wild‐type VZV	20% the HZ risk of wild‐type VZV
Exogenous boosting (protection against HZ after VZV exposure) [4, 14]	100% during 4 years	30% during 20 years 100% during 30 years
Target population catch‐up vaccination [13]	750 000 children, during 7 years	No catch‐up
Vaccine price [30]	Average of list prices (SEK 486)	70%, 50%, 30% of list price average
Vaccine administration cost [13, 29]	SEK 84	50% (SEK 42) 200% (SEK 168)
Added cost of information campaign during first 2 years [46]	Not included	SEK 10 million
*Incidence pre‐vaccination*		
Varicella: incidence (per 1000 population) [2, 33]	Age 0–4 years: 119 Age 5–9 years: 57 Age 10–14 years: 10 Age 15+ years: 1	Not altered
HZ: incidence (per 1000 population) [34, 35]	Age 0–44 years: 1 Age 45–64 years: 3 Age 65–84 years: 7 Age 85+ years: 10	Not altered
Persisting pain following HZ: incidence (% of HZ cases) [35, 43]	Age 0–44 years: 3 Age 45–64 years: 5 Age 65–84 years: 10 Age 85+ years: 14	Not altered
*Healthcare resource use*		
Varicella: Primary care consultation; Pharmaceuticals given; Specialised outpatient consultation; Hospitalisation (% of incident cases) [1, 2, 33]	Age 0–4 years: 9; 0; 2; 0.3 Age 5–9 years: 6; 0; 1; 0.2 Age 10–14 years: 7; 0; 1; 0.3 Age 15+ years: 22; 22; 6; 1.5	Not altered
HZ: Primary care consultation upon onset; Pharmaceuticals given; Specialised outpatient consultation; Hospitalisation (% of incident cases) [2, 34, 35]	Age 0–44 years: 100; 63; 9; 1 Age 45–64 years: 100; 89; 11; 1 Age 65–84 years: 100; 89; 11; 2 Age 85+ years: 100; 89; 11; 6	Not altered
Persisting pain following HZ: Primary care consultation times consultations per patient; Pharmaceuticals given (% of persisting pain cases) [15, 35, 36]	Age 0–44 years: 100 × 5, 58 Age 45–64 years: 100 × 5, 82 Age 65–84 years: 100 × 5, 89 Age 85+ years: 100 × 5, 82	Not altered
*Healthcare unit costs (SEK)*		
Varicella: Primary care consultation; Pharmaceuticals, cost per dose; Specialised outpatient consultation; Hospitalisation [37]	Age 0–14 years: 1280; 0; 4839; 71 723 Age 15–64 years: 1640; 123 5034: 60 154 Age 65+ years: 1937; 123: 5303; 192 923	50%, 200%
HZ: Primary care consultation; Pharmaceuticals, cost per dose; Specialised outpatient consultation; Hospitalisation [37]	Age 0–14 years: 1779; 0; 5134; 61 382 Age 15–64 years: 1843; 123 + 348; 5843; 61 382 Age 65+ years: 1846; 123 + 348; 7077; 75 019	50%, 200%
*Production loss*		
Varicella: Caregiver registered leave (% of incident cases); Days registered; Gross cost per day (SEK) [13, 38]	Age 1–4 years: 40; 3.5; 2109 Age 5–9 years: 45; 3.3; 2109 Age 10–14 years: 19; 3.1; 2109 Age 15+ years: 0; 0; 0	Not altered
Varicella: Days of sick leave adjusted for age‐specific workforce participation; Gross cost per day (SEK) [10, 13]	Age 15–24 years: 1.2; 2109 Age 24–64 years: 3.3; 2109 Age 65–74 years: 0.8; 2109 Age 75+ years: 0; 0; 0	Not altered
HZ: Days of sick leave adjusted for age‐specific workforce participation; Gross cost per day (SEK) [13, 39]	Age 15–24 years: 1.2; 2109 Age 24–64 years: 3.6; 2109 Age 65–74 years: 1.0; 2109 Age 75+ years: 0; 0; 0	Not altered
*QALY loss*		
Varicella: QALY loss primary infection, QALY loss breakthrough infection [41]	Age 0–14 years: 0.0027; 0.0014 Age 15+ years: 0.0038; 0.0019	Not altered
HZ: QALY loss first month; QALY loss persisting pain; Total QALY loss per HZ case [10, 43, 44, 45]	Age 0–44 years: 0.007; 0.231; 0.011 Age 45–64 years: 0.007; 0.221; 0.017 Age 65–84 years: 0.011; 0.207; 0.038 Age 85+ years: 0.011; 0.201; 0.043	Not altered
*Discount rates*		
Discount rate QALYs [23]	3%	0%, 5%
Discount rate costs [23]	3%	0%, 5%

^a^ Average indicative price (SEK 780) [31] minus cost of MMR vaccine (SEK 124) [30] (no vaccine administration cost assumed).

## 3 Results

### 3.1 Base Care Results

A vaccination programme, both without and with catch‐up vaccination of older susceptible children, would be cost‐saving from a societal perspective due to its large impact on reducing caregiver productivity loss. Nearly 80% of the estimated cost‐savings can be attributed to averted caregiver productivity loss, due to a reduced need for parents to be off work to care for children with varicella (see Table 2).

**TABLE 2:** Cost‐effectiveness results (million SEK).

Category	No vaccination (A)	VP without catch‐up (B)	VP with catch‐up (C)	Difference (B‐A)	Difference (C‐A)	Difference (C‐B)
Vaccination programme	0	3837	3837	3837	3837	0
Catch‐up vaccination	0	0	396	0	396	396
Varicella; Healthcare costs	1164	132	48	−1032	−1116	−84
HZ: Healthcare costs	4668	4169	4115	−499	−553	−54
Varicella: Productivity losses	10 035	1007	221	−9027	−9814	−786
HZ: Productivity losses	3584	2773	2704	−811	−880	−69
Total costs (health system)	5832	8138	8396	2306	2563	258
Total costs (societal)	19 451	11 919	11 320	−7532	−8131	−599
Total QALYs	−35 720	−24 374	−23 284	11 346	12 436	1090
Cost/QALY gained (health system perspective)				203 254	206 137	236 697
Cost/QALY gained (societal perspective)				QALYs gained and cost‐saving	QALYs gained and cost‐saving	QALYs gained and cost‐saving

The cost per QALY gained from a health system perspective was estimated at SEK 203 000 from a health system perspective without catch‐up vaccination for older children and SEK 206 000 per QALY gained with catch‐up. The total number of QALYs saved over the 95 years modelled was estimated at 11 300 without and 12 400 with catch‐up respectively.

The catch‐up vaccination of children up to age 12 years on top of the national vaccination programme was cost‐saving per se from a societal perspective. The incremental societal cost‐savings of catch‐up vaccination were estimated at SEK 600 million.

Table 3 presents the discounted cost and cost consequences extracted from the model at different time periods for the two scenarios with and without catch‐up vaccination. The analyses indicate that a varicella vaccination programme both with and without a catch‐up strategy for older susceptible children would be cost‐saving from a societal perspective already in the initial years due to the foreseen reduction in varicella incidence and consequently in averted production loss for caregivers.

**TABLE 3:** Cumulative costs (million SEK).

Cost category	Year 1	Cumulative year 2	Cumulative year 3	Cumulative year 5	Cumulative year 15	Cumulative year 95
*Vaccination programme without catch‐up*						
Cost vaccination programme (a)	65	128	190	307	1238	3837
Varicella: Difference healthcare costs	−7	−20	−42	−85	−332	−1032
HZ: Difference healthcare costs	0	0	1	4	12	−499
Cost difference health system perspective	58	109	148	226	917	2306
Varicella: Difference productivity losses	−35	−136	−331	−670	−2834	−9027
HZ: Difference productivity losses	0	0	0	0	−25	−811
Cost difference societal perspective	23	−27	−183	−444	−1942	−7532
Vaccine doses (‘000)	114	228	342	571	2739	20 997
Varicella: Difference cases (‘000)	−13	−51	−129	−280	−1383	−10 792
Varicella: Difference hospitalisations (‘000)	−0.1	0.22	−0.5	−1	−5	−36
Varicella: Disease burden compared to control (%)	89	78	64	53	22	4
HZ: Difference cases (‘000)	0	0	0	1	1	−905
HZ: Difference hospitalisations (‘000)	0	0	0	0	−0.6	−27
HZ: Disease burden compared to control (%)	100	100	100	101	100	68
*Vaccination programme with catch‐up*						
Cost vaccination programme (a)	65	128	190	307	1238	3837
Cost catch‐up programme (b)	55	119	205	345	395	395
Varicella: Difference healthcare costs	−21	−53	−86	−149	415	1116
HZ: Difference healthcare costs	0	1	3	8	20	−553
Cost difference health system perspective	98	195	312	511	1238	2564
Varicella: Difference productivity losses	−131	−414	−706	−1265	−3615	−9814
HZ: Difference productivity losses	0	1	2	3	−22	−880
Cost difference societal perspective	−33	−219	−392	−750	−2399	−8131
Vaccine doses (‘000)	210	440	714	1215	3486	21 745
Varicella: Difference cases (‘000)	−48	−157	−274	−510	−1687	−11 108
Varicella: Difference hospitalisations (‘000)	−0.2	−0.5	−0.9	−2	−6	−36
Varicella: Disease burden compared to control (%)	60	33	22	14	5	1
HZ: Difference cases (‘000)	0	0	1	2	31	−966
HZ: Difference hospitalisations (‘000)	0	−6	0	0	0.6	−27
HZ: disease burden compared to control (%)	100	101	101	102	101	66

### 3.2 Sensitivity Analyses

From a societal perspective, the vaccination programme remained cost‐saving at all changes in key parameters and therefore, sensitivity analyses are not presented for the societal perspective but only from a health system perspective. Figure 2 provides an overview of how the cost per QALY gained from a health system perspective changed with variation of key parameters. The sensitivity analyses were carried out for a vaccination programme with a catch‐up strategy. The cost per QALY gained remained below SEK 315 000 for all parameter alterations assessed. With a negotiated price reduction of the vaccine price down to approximately SEK 150 per dose, 30% of the list prices at the time of the analysis (which is in line with the current list prices of the MMR‐vaccine) the cost per QALY gained would become as low as SEK 5000.

**FIGURE 2:**
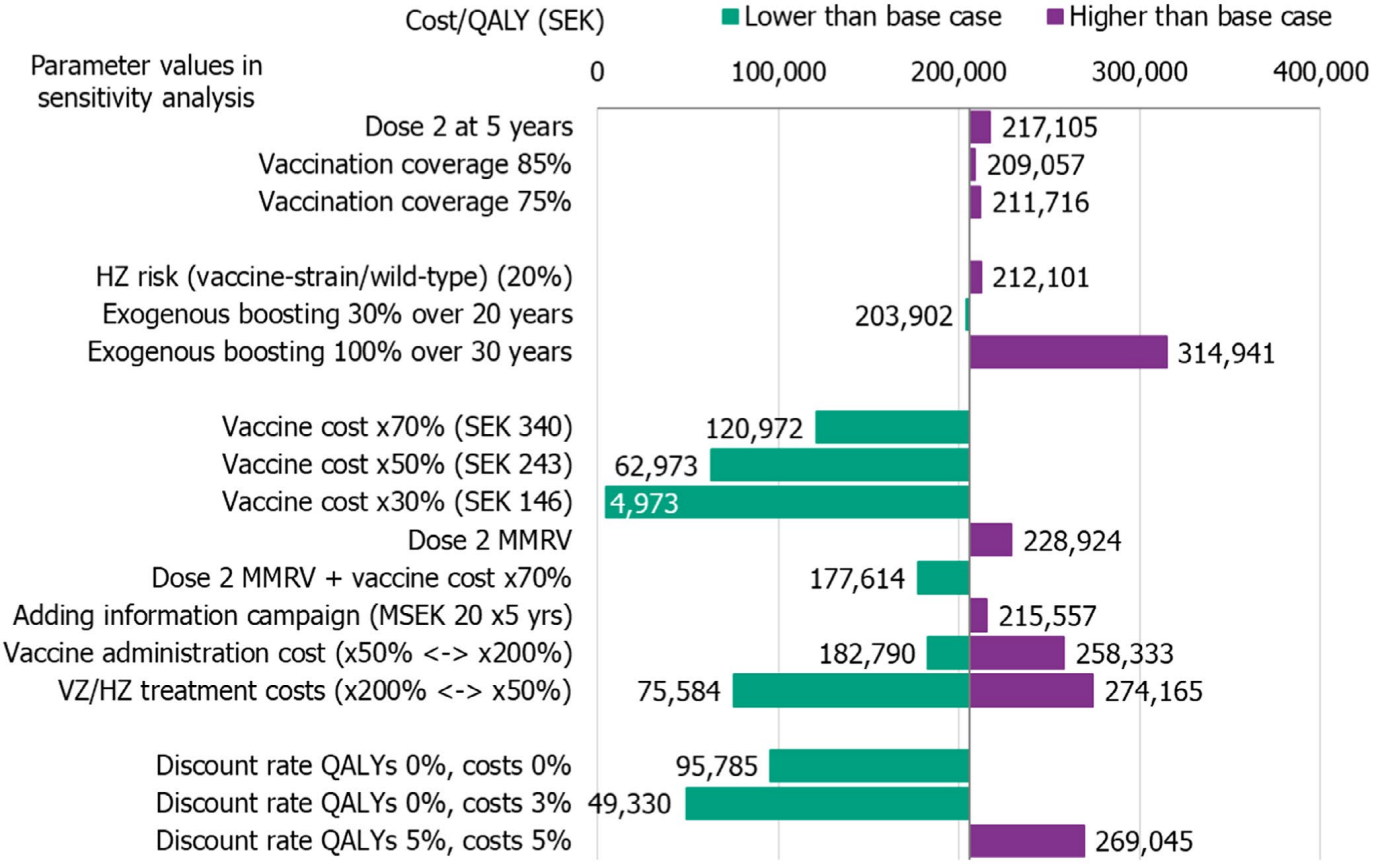
Sensitivity analysis health systems perspective (*y*‐axis, vaccination programme with catch‐up: SEK 206 137).

## 4 Discussion

Our analyses indicate that a national varicella vaccination programme in Sweden, without or with a catch‐up vaccination of older susceptible children, is cost‐saving from a societal perspective. The cost‐savings are primarily due to a reduction in caregiver productivity losses. From a health system perspective, the cost per QALY was estimated at approximately SEK 200 000 (EUR 18 000). The additional catch‐up strategy is cost‐saving in itself from a societal perspective, with a cost per QALY of approximately SEK 240 000 (EUR 21 000) from a health system perspective. Since catch‐up vaccination will be offered to susceptible children based on recall of care‐providers of past infection in the child, this estimation is contingent on that the children who are indeed susceptible are the ones captured in a catch‐up strategy. The calculations are based on current list prices of the vaccines. These costs can be expected to be reduced in a situation with national tenders for the vaccines.

We have compared varicella vaccination with a situation with no vaccination. However, in some parts of Sweden, particularly in the major cities, private vaccination rates have increased in recent years. Sub‐optimal vaccine coverage obtained through private vaccinations may drive infection to occur at higher ages. A national vaccination programme with sufficiently high coverage in combination with a catch‐up strategy would avert this risk [47].

To date, Finland and Iceland are the only Nordic countries that have introduced varicella vaccination as part of their national child vaccination programmes. A cost‐effectiveness analysis conducted by the National Public Health Institute of Finland in 2008 estimated a cost per QALY gained of EUR 4900 for a two‐dose vaccination programme and of EUR 8700/QALY gained for a programme including catch‐up vaccination for children up to 12 years of age for a health system perspective [48]. In Iceland, a varicella vaccination programme was estimated to be cost‐saving from a societal perspective [49]. Economic evaluations of universal varicella vaccination in Denmark and Norway estimated that varicella vaccination would result in a cost per QALY gained between EUR 3700 and 5900 from a societal perspective and EUR 18 000–20 300 from a health system perspective in Denmark [50], and cost‐saving from both a societal and health system perspective in Norway [51]. Thus, all economic evaluations of varicella vaccination programmes in the Nordic countries indicated that programmes would be cost‐effective according to commonly applied thresholds or cost‐saving.

## 5 Limitations

A varicella vaccination model is a simplified version of reality. However, our model prediction that high coverage universal varicella vaccination will make endemic varicella essentially disappear after a few years is upheld by the experience of countries that have initiated such general programmes.

In our model, the HZ risk after vaccination was based on the relative incidence in younger individuals up to 18 years of age in the US routine varicella vaccination programme [21, 22]. Longer follow‐up data is needed to assess the risk of HZ following varicella vaccination in infancy throughout the life course. Therefore, modelling becomes quite hypothetical and without real predictive power for HZ prevention. Furthermore, one of the main reasons for uncertainty about the introduction of general varicella vaccination has been the possible effect that a decline in circulating VZV in a population would have on HZ incidence due to reduced exogenous boosting. However, recent observational data, in accord with our modelling results, seem to indicate that these effects are not significant [22, 52].

## 6 Conclusion

To conclude, our analyses suggest that the inclusion of varicella vaccination in the national vaccination programme for children in Sweden would be a cost‐saving strategy from a societal perspective. From a health system perspective, the cost would be approximately SEK 200 000 (EUR 18 000) per QALY gained with the current list prices of the vaccines. Together with a catch‐up vaccination offer for older susceptible children, national varicella vaccination would result in a fast decline in circulating VZV in Sweden.

## Author Contributions

Frida Kasteng: conceptualization, methodology, formal analysis, investigation, validation, writing – original draft. Gianpaolo Scalia Tomba: methodology, formal analysis, writing – review and editing. Annika Ersson: conceptualization, writing – review and editing. Ellen Wolff: methodology, formal analysis, investigation, writing – review and editing. Sören Andersson: conceptualization, writing – review and editing. Kari Johansen: conceptualization, writing – review and editing.

## Funding

The study was supported entirely by the Public Health Agency of Sweden, Solna, Sweden.

## Conflicts of Interest

The authors declare no conflicts of interest.

## Data Availability

The data that support the findings of this study are available from the corresponding author upon reasonable request.
